# Prognostic comparison of tissue Doppler indices of diastolic dysfunction and cardiac biomarkers in septic shock

**DOI:** 10.1186/cc14245

**Published:** 2015-03-16

**Authors:** V Karali, V Voutsas, N Loridas, M Konoglou, M Papaioannou, A Alexiou, V Hatsiou, C Fitili, M Bitzani

**Affiliations:** 1 Papanikolaou Hospital, Thessaloniki, Greece

## Introduction

Diastolic dysfunction as evaluated by tissue Doppler imaging (TDI), particularly by E/E' ratio (peak early diastolic transmitral velocity/peak early diastolic mitral annular velocity) and mitral annular E'-wave, is common and crucial in critical illness. Our prospective observational study assessed the prognostic significance of TDI variables versus cardiac biomarkers, B-type natriuretic peptide (BNP), troponin-T (TnT) and investigated determinants of plasma BNP rise, in septic shock.

## Methods

Twenty-seven mechanically ventilated adult patients admitted to our ICU were evaluated within 72 hours of evolving septic shock. Patients underwent two transthoracic echocardiographies within 72 hours of the onset of septic shock: shortly after diagnosis and 24 hours later (confirmatory), alongside relevant measurements of cardiac biomarkers. Peak mitral inflow E and A velocity waves were recorded using pulsed-wave Doppler at the mitral valve tips from the apical four-chamber view, peak early (E') and late (A') diastolic myocardial velocities were obtained by TDI at the septal mitral annulus in the apical four-chamber view. E/E' was calculated. *P *≤0.01 was reported as statistically significant.

## Results

Mean ± SD APACHE II score was 21.22 ± 7.28, mean ± SD admission SOFA score 10.25 ± 2.76. Hospital mortality was 55%. Nonsurvivors had increased E/E' (15.56 ± 1.48; *P *<<0.00001) and reduced E' 6.32 ± 0.68 cm/second (*P *<<0.00001) compared with survivors, who exhibited inverse correlations with an E/E' significantly lower (9.30 ± 2.88) and higher E' (9.01 ± 0.85 cm/second). In contrast, BNP and TnT levels displayed remarkably lower statistical significance in nonsurvivors (*P *= 0.005, *P *= 0.007 respectively). The ROC curves had an area under the curve of 0.98 for the E', and 0.92 for the E/E'. Vasopressor management (noradrenaline dose) (*P *= 0.0001), fluid balance (*P *<0.001) and E/E' (*P *= 0.00004) were independent predictors of plasma BNP concentration.

## Conclusion

Diastolic dysfunction as evaluated by E/E' and E' constitutes a major independent predictor of outcome in septic shock, compared with cardiac biomarkers, suggesting that echocardiographic techniques assessing diastolic dysfunction in sepsis may replace cardiac biomarkers for mortality prediction. Fluid balance, vasopressor management and diastolic dysfunction are independent predictors of BNP elevation in septic shock. Our findings should be confirmed by an extended prospective study.

